# A Method for Predicting Coal-Mine Methane Outburst Volumes and Detecting Anomalies Based on a Fusion Model of Second-Order Decomposition and ETO-TSMixer

**DOI:** 10.3390/s25113314

**Published:** 2025-05-24

**Authors:** Qiangyu Zheng, Cunmiao Li, Bo Yang, Zhenguo Yan, Zhixin Qin

**Affiliations:** 1 College of Arts, Xi’an University of Science and Technology, Xi’an 710054, China; y13572288566@163.com (Q.Z.); 13303916403@163.com (C.L.); 2 College of Safety Science and Engineering, Xi’an University of Science and Technology, Xi’an 710054, China; 24220214180@stu.xust.edu.cn (B.Y.); yanzg@xust.edu.cn (Z.Y.)

**Keywords:** TSMixer, CEEMDAN-Kmeans-VMD decomposition, exponential triangle optimization algorithm, anomaly detection

## Abstract

The ability to predict the volume of methane outbursts in coal mines is critical for the prevention of methane outburst accidents and the assurance of coal-mine safety. This paper’s central argument is that existing prediction models are limited in several ways. These limitations include the complexity of the models and their poor ability to generalize. The paper proposes a methane outburst volume-prediction and early-warning method. This method is based on a secondary decomposition and improved TSMixer model. First, data smoothing is achieved through an STL decomposition–adaptive Savitzky–Golay filtering–reconstruction framework to reduce temporal complexity. Second, a CEEMDAN-Kmeans-VMD secondary decomposition strategy is adopted to integrate intrinsic mode functions (IMFs) using K-means clustering. Variational mode decomposition (VMD) parameters are optimized via a novel exponential triangular optimization (ETO) algorithm to extract multi-scale features. Additionally, a refined TSMixer model is proposed, integrating reversible instance normalization (RevIn) to bolster the model’s generalizability and employing ETO to fine-tune model hyperparameters. This approach enables multi-component joint modeling, thereby averting error accumulation. The experimental results demonstrate that the enhanced model attains RMSE, MAE, and R^2^ values of 0.0151, 0.0117, and 0.9878 on the test set, respectively, thereby exhibiting a substantial improvement in performance when compared to the reference models. Furthermore, we propose an anomaly detection framework based on STL decomposition and dual lonely forests. This framework improves sensitivity to sudden feature changes and detection robustness through a weighted fusion strategy of global trends and residual anomalies. This method provides efficient and reliable dynamic early-warning technology support for coal-mine gas disaster prevention and control, demonstrating significant engineering application value.

## 1. Introduction

Coal is recognized as one of the world’s primary energy sources, and during the ongoing energy transition, it is anticipated to maintain its pivotal role in the global energy mix. The primary objective of coal-mine production is to ensure safety. Among the various types of production safety accidents in coal mines, gas-related accidents are the primary cause of casualties [1,2,3]. As coal mines increasingly transition to deep mining methods, there is an escalating risk of coal and gas outburst disasters. These disasters pose a grave threat to the safety of coal-mine operations. In the period preceding gas-related disasters, anomalous fluctuations in gas concentration levels frequently manifest [4]. Consequently, methane concentration can function as an early indicator of the potential for coal and methane outbursts. The ability to predict methane concentration is of paramount importance for the prevention and control of methane accidents and the assurance of safe production in coal mines.

The crux of gas prediction lies in the revelation of the nonlinear characteristics inherent in gas data fluctuations through the employment of models to analyze historical time-series data. Gas prediction methods can be categorized into three distinct types: traditional methods, machine learning methods, and deep learning methods. A variety of conventional prediction methods are employed, including autoregressive integrated moving average (ARIMA) and autoregressive conditional heteroskedasticity (GARCH), along with their enhanced models, such as autoregressive moving average with exogenous disturbances (ARMAX). However, the conventional methods employed in this field necessitate data that remain constant and are unable to effectively adapt to scenarios involving nonlinear sudden change. Machine learning methodologies encompass Support Vector Machines (SVMs), random forests (RFs), and Backpropagation Neural Networks (NNs), among other approaches [5,6]. In comparison with conventional methodologies, these approaches demonstrate enhanced robustness and specific nonlinear mapping capabilities. Nevertheless, they are not fully equipped to capitalize on the temporal dependency inherent in the problem. Consequently, deep learning algorithms have been extensively employed in the domain of methane outburst volume time-series prediction. Karacan and C. Özgen [7] proposed an artificial neural network (ANN) based on a multi-layer perceptron (MLP) to enhance the precision of methane concentration forecasting in gas venting holes (GVHs) within abandoned mine areas. Krzemień and Alicja [8] investigated the potential of the multivariate adaptive regression spline (MARS) method to predict temperature in the context of underground coal gasification (UCG) processes in production mines, with the aim of preventing fires. Dey, Prasanjit Chaulya, and others [9] proposed a CNN-LSTM prediction model deployed on an IoT sensor hybrid system with the objective of enhancing safety in underground production. Niu Yue Wang, Li Enyuan, and others [10] established a bilateral EP inversion model for coal seams affected by mining activities. This model is capable of accurately monitoring the spatial distribution of coal and gas outburst hazard zones, and the researchers conducted field applications. Wang et al. [11] proposed an IDBO-BPNN coal-body methane permeability prediction model capable of producing accurate predictions of coal gas permeability. Gao Yifei, Zhang Xiaohang, and others [12] proposed a parallel-structure graph convolutional encoder–decoder (GCN-ED) network, which can uniformly train all sensors in the working face to improve the efficiency and accuracy of methane concentration prediction. Xue Haiteng, Gui Xiaohong, and others [13] employed the Attention–TCN model to achieve a satisfactory prediction of nitrogen injection volume changes in coal mines, replacing pure gas volume changes in coal layers. The model’s effectiveness was verified using ten-fold cross-validation results. Wang Yanping, Qin Zhixin, and others [14] proposed an interpretable AFT–Transformer–SVM model, which demonstrates high prediction accuracy for methane outburst volume. Huang Yaping, Yan Lei, and others [15] employed the VMD decomposition method to perform secondary decomposition on the trend components obtained from EMD. They then combined this with an LSTM model to predict coal seam thickness, achieving high prediction accuracy. Xu Ningke, Wang Xiangqian, and others [16] developed an IWOA-LSTM-CEEMDAN model, enhancing the precision of the multi-step prediction of methane outburst volume. Ji Peng, Shi Shiliang, and others [17] optimized the hyperparameters of the BiLSTM model using the HPO algorithm, enabling the early warning of methane outbursts and guiding safe coal-mining operations. Yu Kai, Zhou Lujie, and others [18] proposed a method for predicting and issuing early warnings regarding methane risks in coal mines. This method is based on the fusion of behavior information, and it involves the construction of a model for coal mine safety situation awareness. This approach led to substantial enhancements in the precision of methane data prediction, risk value prediction, and safety situation value prediction. Researchers have achieved significant advancements in methane outburst prediction methods based on deep learning. These advancements involve the integration of multi-domain algorithms to optimize or establish models.

However, in the context of engineering practice, most coal mines are unable to provide detailed data on the relevant parameters influencing methane outburst volume at the tunneling working face. Additionally, there is the problem of insufficient effective sample sizes in monitoring data. Conventional prediction models demonstrate suboptimal generalizability, impeding their applicability across diverse mining environments. Moreover, these models are incapable of fulfilling demands for real-time methane concentration forecasting. In order to address these issues and improve the accuracy of methane outburst prediction, this study analyzes methane outburst data under conditions with limited data indicators for tunneling workfaces. A multi-model TSMixer based on quadratic decomposition has been improved to achieve the real-time prediction of methane outbursts at tunneling workfaces. Furthermore, the implementation of anomaly detection methods facilitates the generation of warning outputs based on prediction results, with the objective of establishing an efficient and reliable dynamic warning model for methane outbursts.

## 2. Materials and Methods

The central objective of this paper is to propose a real-time methane prediction model based on an enhanced TSMixer. The model utilizes a smoothing method comprising STL decomposition, adaptive Savitzky–Golay filtering, and reconstruction to process interpolated data. This process reduces data complexity and enhances methane concentration prediction accuracy. Subsequently, the Complete Ensemble Empirical Mode Decomposition with Adaptive Noise (CEEMDAN) + K-means clustering + variational mode decomposition (VMD) data decomposition method was employed. The decomposed intrinsic mode function (IMF) components serve as the input for the TSMixer model. TSMixer has been enhanced by the incorporation of the reversible instance normalization (RevIn) method, which has been demonstrated to improve the model’s generalization capability. Offline data were utilized as the training set for model training. The ETO algorithm was employed to optimize the parameters of VMD and TSMixer, and the optimal parameter set was subsequently applied for model training. The model that was generated was applied to an online prediction in a mine production environment. Consequently, a methane concentration anomaly detection method was proposed, which utilizes STL decomposition (Seasonal and Trend Decomposition using Loess) and the concept of ‘lonely forest’. This approach offers a viable solution for mitigating the risk of coal and methane outburst accidents.

**Figure 1:**
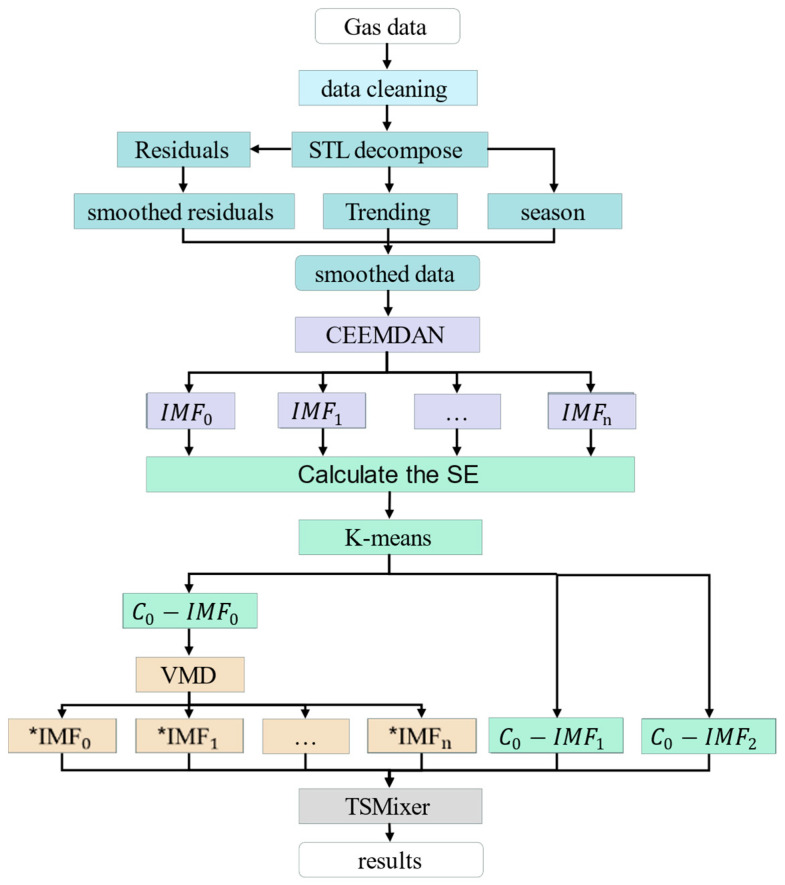
The overall framework for gas concentration prediction.

As illustrated in Figure 1, the overall framework for gas concentration prediction is composed of several interconnected components. Initially, data preprocessing is conducted. The data undergo a series of processing steps, including smoothing using STL decomposition, adaptive Savitzky–Golay filtering, and reconstruction. Subsequently, the data undergo decomposition utilizing CEEMDAN + K-means + VMD, and the resulting IMF is collectively integrated into the enhanced TSMixer model for prediction.

### 2.1. Data Preprocessing

#### 2.1.1. Data Cleaning

The acquisition of comprehensive and reliable methane data serves as a foundational element for the prediction of methane outburst volumes. During the collection, transmission, and storage of safety monitoring data at the tunneling workface, the original methane data may experience local data loss and abnormal fluctuations due to complex underground conditions, such as dust, moisture, and electromechanical interference. Consequently, the process of incorporating data into a prediction model necessitates the preliminary management of outliers and missing values to ensure the validity of the data.

In order to ensure data continuity, it is necessary to perform linear interpolation on the data [19]. The fundamental formula of the linear interpolation method is

(1) $$f_{\left(x\right)}=y_{0}+\frac{\left(x-x_{0}\right)\left(y_{1}-y_{0}\right)}{x_{1}-x_{0}}$$

In Equation (1), $f_{\left(x\right)}$ represents the estimated result of the missing value, and $x_{0}$ and $y_{0}$ represent the independent- and dependent-variable values of the known points adjacent to the left of the missing value. Likewise, $x_{1}$ and $y_{1}$ represent the independent- and dependent-variable values of the known points adjacent to the right of the missing value. Finally, x represents the independent-variable value at the position to be interpolated.

The IQR (interquartile range) method is employed to identify outliers, ensuring the reliability of the data. The interquartile range (IQR) is defined as the difference between the third quartile (Q3) and the first quartile (Q1), with the range of the middle 50% of the dataset serving as the primary indicator. The outlier boundaries are delineated and outliers are identified using this standard, which is based on a predefined expansion factor of 1.5.

#### 2.1.2. Data Smoothing

The smoothing method employed in this paper utilizes a multi-stage decomposition–filtering–reconstruction framework to achieve the smoothing of non-stationary time series. The technical principles and implementation process can be summarized as follows:

Step 1: STL Decomposition [20] and Extraction of Deterministic Components

This decomposition process complies with Cramer’s theorem regarding the mathematical expression of time series as a superposition of deterministic and random components:

(2) $$X_{t}=T_{t}+S_{t}{+R}_{t}$$

In Equation (2), $X_{t}$ represents the original methane concentration signal; $T_{t}$ represents the trend component; $S_{t}$ represents the seasonal component; and $R_{t}$ represents the residual component, i.e., the random fluctuations not explained by the trend and seasonal components, including high-frequency noise and irregular shocks.

Step 2: Residual Dynamic Smoothing and Denoising

The analysis of the residual sequence reveals significant non-stationary characteristics and random fluctuations, which are not conducive to the prediction of time series models. Consequently, the adaptive Savitzky–Golay filter [21] is employed to process the residuals, thereby enhancing the model’s resilience against random disturbances.

The present paper implements a window dynamic adjustment mechanism, which, through dynamic windows and parameterized design, renders the method applicable to different mine conditions. In the context of high-methane mines, for instance, residual fluctuations are pronounced, necessitating the augmentation of window size (window_ratio adjusted to 0.2 or higher) to mitigate noise. Conversely, in low-methane mines, a more diminutive window can be employed to preserve intricate details. The length of the window is dynamically calculated based on the window_ratio and forced to an odd number to ensure filter symmetry and avoid phase shifts. The minimum window size is limited to 3 to prevent overfitting. This method achieves third-order polynomial fitting, balancing the need for both smoothness and shape retention, and effectively preserves potential short-term trends in the decomposition residuals (such as gradual changes in local methane accumulation). Interpolation compensation, also referred to as ‘interp mode’, is a method that has been demonstrated to circumvent the edge data truncation issue that is inherent to traditional sliding window methods. This approach serves to augment the robust design of STL, thereby enhancing its overall functionality and reliability. This approach addresses the information loss issues that may arise in traditional difference methods (e.g., ARIMA), better preserves local features of the sequence compared to first-order differences, and is more suitable for scenarios with nonlinear fluctuations in gas concentration.

Step 3: Sequence Reconstruction and Postprocessing

This objective is primarily accomplished through the application of component superposition and alignment, thereby ensuring a smooth output. Initially, the processed trend, seasonal, and smoothed residual components are recombined to form a smoothed sequence. Subsequently, the original timestamps are aligned, and missing edges caused by window sliding are addressed to ensure that the output sequence matches the input length. The reconstructed stationary sequence is more suitable as input for deep learning models, reducing the risk of gradient anomalies or overfitting caused by non-stationarity. Non-stationary processes can be suppressed while preserving the certainty information of the trend and seasonal components. This approach prevents prediction degradation caused by over-stationarization.

### 2.2. Mode Decomposition

#### 2.2.1. CEEMDAN

CEEMDAN [22] is a multimodal decomposition technique developed by Torres et al. based on the Empirical Mode Decomposition (EMD) and Ensemble Empirical Mode Decomposition (EEMD) algorithms. With its adaptive noise mechanism, staged decomposition strategy, and efficient noise resistance, it has become an important tool for analyzing non-stationary time series. Not only does it largely solve the problems of mode aliasing and noise residue in the EMD and EEMD processes, but its decomposition process integrity also ensures the accurate reconstruction of the original signal, while also improving computational efficiency. CEEMDAN achieves this by adaptively adding and averaging noise after each stage of intrinsic mode function (IMF) decomposition, thereby progressively eliminating high-frequency noise interference on low-frequency components and precisely separating different frequency components. This effectively suppresses the modal aliasing issues commonly encountered in traditional methods. Additionally, the iterative averaging process further reduces the impact of random noise on the decomposition results, making it suitable for processing noisy non-stationary signals. Therefore, we employ the CEEMDAN algorithm to decompose the original methane concentration sequence to reduce its complexity and volatility.

The implementation process of the CEEMDAN algorithm is as follows:

Step 1: Inject white noise with a u distribution N times into the original signal h(t) to construct the signal to be decomposed.

(3) $$h^{i}(t)=h(t)+\sigma_{0}\cdot \varepsilon^{i}(t);i=1,2,...,N$$

In Equation (3), t is the data sampling time, $h^{i}(t)$ is the new signal formed after noise injection, h(t) is the original signal, $\sigma_{0}$ denotes the initial adaptive weighting coefficient, and $\varepsilon^{i}(t)$ denotes the noise sequence added at the ith iteration.

Step 2: Perform EMD on the data sequence after noise injection to obtain the extracted first-order modal components. Calculate the mean of the modal components after N noise superpositions, as shown in Equation (4), and calculate the current-order residual using Equation (5).

(4) $$F_{1}=\frac{1}{N}\cdot \sum_{i=1}^{N}F_{1}^{i}$$

(5) $$r_{1}\left(t\right)=h\left(t\right)-F_{1}$$

Step 3: Inject noise into the *k*th (*k* = 2, 3, …, N) residual component obtained from the decomposition, and proceed to the next step of decomposition and calculation.

(6) $$F_{k}=\frac{1}{N}\cdot \sum_{i=1}^{N}E_{1}\left[r_{k-1}\left(t\right)+\sigma_{K-1}\cdot E_{K-1}(\varepsilon^{i}(t))\right]$$

(7) $$r_{k}\left(t\right)=r_{k-1}\left(t\right)-F_{k}$$

In Equation (6), $\sigma_{K-1}$ are noise weighting coefficients that decrease with order, and $F_{k}$ are *k*-order modal components.

Step 4: Repeat Step 3 until the residual component cannot be further decomposed using EMD. The algorithm terminates, and the original signal sequence h(t) is decomposed into *N* modal components and a residual term R(t).

(8) $$h\left(t\right)=\sum_{i=1}^{N}F_{i}+R(t)$$

CEEMDAN decomposition may generate a large number of IMF features with high dispersion, leading to difficulties in parameter optimization during the secondary decomposition of VMD. Additionally, high-frequency IMFs often contain random noise, and when unclustered, the noise components may directly enter the prediction model. Therefore, this paper employs K-means clustering to integrate IMFs. Through the use of sample entropy (SE) clustering, IMFs can be categorized into three classes: high-frequency noise, mid-frequency periodic, and low-frequency trend. This reduces the complexity of subsequent processing while enhancing the model’s generalization ability and robustness.

#### 2.2.2. K-Means

The k-means [23] algorithm is a distance-based clustering algorithm that is widely used in scientific and industrial fields due to its simple structure and fast convergence speed. Its principle is to first divide the sample points into k clusters based on their distances to the cluster centers, and then iteratively update the clusters until the distances between the samples and their cluster centers are minimized.

This study employs the k-means clustering algorithm to perform feature fusion on the IMF generated by CEEMDAN decomposition. The specific process is as follows:

Step 1: Calculate the sample entropy of each IMF to construct feature vectors for quantifying signal complexity. The SampEn calculation formula is as follows:

(9) $$SampEn\left(m,r,N\right)=-\ln\frac{A}{B}$$

In Equation (9), A  represents the total number of similar vectors in the m+1  dimensional space; B represents the total number of similar vectors in the m dimensional space; m  represents the dimension of the vector; r  is the threshold for determining vector similarity; and N is the sequence length.

Step 2: Set the number of clusters K = 3 and use the k-means strategy to initialize the cluster centers to optimize algorithm stability.

Step 3: Iteratively assign IMFs to the nearest cluster based on the principle of minimizing the Euclidean distance, and perform the linear superposition of modes in the same cluster to generate fusion components.

Step 4: Screen high-complexity fusion components based on entropy value sorting, and use VMD secondary decomposition to effectively suppress noise interference.

#### 2.2.3. VMD

The VMD [24] method is a non-recursive signal decomposition method that effectively addresses the issues of mode aliasing and high-frequency signal loss in traditional EMD methods. It offers higher robustness and decomposition accuracy and is suitable for non-stationary time series. By iteratively searching for the optimal solution of the constrained variational mode, the original signal can be decomposed into a series of intrinsic mode functions (IMFs) with different frequency ranges, thereby minimizing the total bandwidth of all modal-component center frequencies and obtaining the corresponding modal-component signals and related parameters.

Equation (10) is the amplitude-modulated and frequency-modulated component mode function, where $A_{k}\left(t\right)$ is the amplitude of $u_{k}\left(t\right)$; $\varphi_{k}\left(t\right)$ is the instantaneous phase, rad.

(10) $$u_{k}\left(t\right)=A_{k}\left(t\right)cos[\varphi_{k}(t)]$$

The VMD process is the solution process of the variational problem, and the constructed constraint variational model is shown in Equation (11).

(11) $$\left\{\begin{array}{c} \underset{\left\{u_{k}\right\}\{\omega_{k}\}}{\min}\left\{\sum_{k=1}^{K}{\left\|\partial_{t}\left[\left(\delta (t)+\frac{j}{\pi t}\right)*u_{k}(t)\right]e^{-j\omega_{k}(t)}\right\|}_{2}^{2}\right\}\\ s.t.\;\sum_{k=1}^{K}u_{k}\left(t\right)=f(t) \end{array}\right.$$

In Equation (11), $\partial_{t}$ denotes partial derivatives; ∗ denotes convolution; ${∥\cdot ∥}_{2}^{2}$ denotes the vector norm squared; f(t) denotes the original methane concentration signal; $\delta \left(t\right)$ denotes the pulse function; and K denotes the number of modes to be decomposed.

Introducing a penalty term α and a Lagrange multiplier λ(t) to construct the Lagrange function, we obtain

(12) $$L\left(\left\{u_{k}\right\},\left\{\omega_{k}\right\},\lambda \left(t\right)\right)=\;\alpha \sum_{k=1}^{K}{\left\|\partial_{t}\left[\left(\delta (t)+\frac{j}{\pi t}\right)*u_{k}(t)\right]e^{-j\omega_{k}(t)}\right\|}_{2}^{2}+\;{\left\|f\left(t\right)-\sum_{k=1}^{K}u_{k}(t)\right\|}_{2}^{2}+\langle \lambda \left(t\right),f\left(t\right)-\sum_{k=1}^{K}u_{k}(t)\rangle$$

The expressions for iteratively updating the saddle point, modal components, and center frequency using the alternating direction method of multipliers to solve Equation (13) are as follows:

(13) $$U_{k}^{n+1}\left(\omega \right)=\;\frac{F\left(\omega \right)-\sum_{j\neq k}U_{j}\left(\omega \right)+\frac{Τ(\omega )}{2}}{1+2\alpha {\left(\omega -\omega_{k}\right)}^{2}}$$

(14) $$\omega_{k}^{n+1}=\frac{\int_{0}^{\infty }\omega {\left|U_{k}\left(\omega \right)\right|}^{2}d\omega }{\int_{0}^{\infty }{\left|U_{k}\left(\omega \right)\right|}^{2}d\omega }$$

In Equation (13), $F\left(\omega \right)$, $U_{k}\left(\omega \right)$, and Τ(ω) are the corresponding frequency-domain expressions for f(t), $u_{k}(t)$, and λ(t), respectively; n is the number of iterations; and ω denotes the frequency.

The decomposition performance of VMD depends on the pre-set number of components k and the penalty factor. A large k value leads to information dilution, making it difficult for subsequent models to capture effective patterns. A large value may result in the loss of frequency band information, while a small value or k value may exacerbate modal overlap. The method of pre-setting empirical values cannot address the scenarios of gas outbursts in different mining areas and cannot determine the optimal parameter combination. Therefore, this study proposes the optimization of VMD parameters using the ETO algorithm to achieve the goal of effectively decomposing gas concentration signals.

#### 2.2.4. ETO Algorithm

The exponential–trigonometric optimization (ETO) algorithm [25] is a novel meta-heuristic algorithm proposed in 2024. Its core mechanism leverages the synergistic interaction between exponential functions and trigonometric functions to efficiently adjust the positions of search agents, thereby establishing a dynamic balance between global search and local exploration. The following sections present the mathematical modeling and analysis of the algorithm’s key components:

(1) Constraint Exploration Strategy

By dynamically contracting the boundaries of the search space, the algorithm reduces the wasting of time and computational resources while ensuring that no optimal solutions are overlooked. The upper- and lower-limit update formulas are as follows:

(15) $$\begin{array}{c} U_{p_{i}}=r_{1}\times \left(1-\frac{t}{Max\_ter}\right)\times \left|r_{2}\times X_{j}^{best}-X_{j}^{s}\right|+X_{j}^{best}\\ Low_{i}=-r_{1}\times \left(1-\frac{t}{Max\_ter}\right)\times \left|r_{2}\times X_{j}^{best}-X_{j}^{s}\right|+X_{j}^{best} \end{array}$$

In Equation (15), $U_{p_{i}}$ and $Low_{i}$ represent the boundaries of the search space; $r_{2}$ and $r_{1}$ are coefficients randomly selected from [0, 1]; $X_{j}^{best}$ stores the *j*th position of the optimal solution; $X_{j}^{s}$ is the position of the suboptimal solution in the *j*th criterion; and Max_ter is the total number of iterations.

(2) Initialization and Population Generation

The population is initialized using a uniform random distribution:

(16) $$x_{i,j}={low}_{j}+rand\left(\;\right)\cdot \left({up}_{j}-{low}_{j}\right)$$

In Equation (16), $rand\left(\right)$ generates a uniform random number in the range of [0, 1], ensuring that the initial solution covers the multidimensional search space.

(3) Exploration Mechanism

The step length is dynamically adjusted through the index decay coefficient and cosine oscillation term to prevent convergence to local optima. Wide-area exploration is focused on in the early stages, with a shift to detailed development in the later stages.

(17) $$X_{t+1}^{i,j}=\left\{\begin{array}{ll} X_{j}^{best}+rand\left(\;\right)\times a_{1}\times \left|X_{j}^{best}-X_{t}^{i,j}\right|,\;q_{1}>0.5 & \\ X_{j}^{best}-rand\left(\;\right)\times a_{1}\times \left|X_{j}^{best}-X_{t}^{i,j}\right|,\;q_{1}<0.5 & \end{array}\right.$$

In Equation (17), $X_{t+1}^{i,j}$ and $X_{t}^{i,j}$ represent the jth position of the ith solution in the next and current iterations, respectively; *t* is the current iteration label; and $a_{1}$ is the weight coefficient.

(4) Development Mechanism

As iteration proceeds, the intensity of local exploration increases, and Equation (18) controls the strategy of strengthening search in the most favorable region of the search space:

(18) $$X_{t+1}^{i,j}=X_{t}^{i,j}+c\times \left|rand\left(\;\right)\times \alpha_{2}\times X_{j}^{best}-X_{t}^{i,j}\right|,c=exp\left[tan\left(\left|\frac{d_{1}}{d_{2}}\right|\right)\right]$$

Index attenuation and sine-wave fluctuations are combined to achieve dynamic focusing, enhancing the ability to update the optimal location and balancing convergence speed and stability.

(5) Exploration–Development Adaptive Transition

A new transition mechanism has been developed to match and optimize the efficiency of the ETO algorithm:

(19) $$CM=0.01\times rand\left(\;\right)\times {\left[sqrt\left(\frac{t}{Max\_ter}\right)\right]}^{tan\left(\frac{d_{1}}{d_{2}}\right)}$$

In Equation (19), When CM is greater than or equal to 1, ETO is in exploration mode; otherwise, it switches to development mode.

### 2.3. Time Series Model

As the coal-mining face advances, the methane content released into the air from the coal seam fluctuates significantly, exhibiting pronounced nonlinear variations in methane data. These fluctuations can greatly impact the accuracy of methane outburst predictions. TSMixer [26] is a multi-layer perceptron (MLP)-based multimodal time-series prediction model proposed by Google in September 2023. It achieves more flexible multi-scale feature fusion through the MLP, avoiding the complexity and high computational demands of traditional recurrent neural networks (RNNs) and attention mechanisms (such as Transformers), while enhancing the ability to capture sudden high-frequency changes in non-stationary data. Therefore, in engineering practice, the TSMixer model is more suitable for real-time methane prediction scenarios.

TSMixer adopts a hierarchical stacked hybrid layer structure, where each hybrid layer consists of two modules: time mixing and feature mixing. Through the alternating operations of time mixing and feature mixing, it can decompose complex temporal patterns in methane data.

The time-mixing MLP is responsible for mixing information along the time steps. It first transposes the rows and columns of the input matrix to apply fully connected layers along the time steps, capturing the temporal dependencies in methane data. Nonlinear activation functions are then applied to introduce nonlinearity, followed by a dropout layer to reduce overfitting. Finally, the first residual connection is established to retain the original temporal patterns and prevent information loss in deep networks.

(20) $$Z=Dropout\left({\left[\sigma \left({\left(LayerNorm\left(X\right)\right)}^{T}\cdot W_{t}+b_{t}\right)\right]}^{T}\right)+X$$

In Equation (20), $X\in R^{B\times T\times d}$ is the input tensor, B is the batch size, T is the time step, and d is the channel dimension; σ is the activation function; and $W_{t}$ and $b_{t}$ denote the parameters of the fully connected layer.

The feature-mixing MLP is responsible for information mixing at the feature dimension level. It consists of two fully connected layers: the first layer extracts interactions between features, and the second layer performs further feature transformation, also using nonlinear activation functions and dropout. Finally, the results are fused with time-series data to maintain the stability of cross-variable relationships.

(21) $$Y=Dropout\left(\sigma \left(LayerNorm\left(Z\right)\cdot W_{1}+b_{1}\right)\cdot W_{2}+b_{2}\right)+Z$$

In Equation (21), Z represents the output of the time-hybrid MLP; $W_{1}$ and $b_{1}$ represent the parameters of the feature expansion layer; and $W_{2}$ and $b_{2}$ represent the parameters of the feature compression layer.

**Figure 2:**
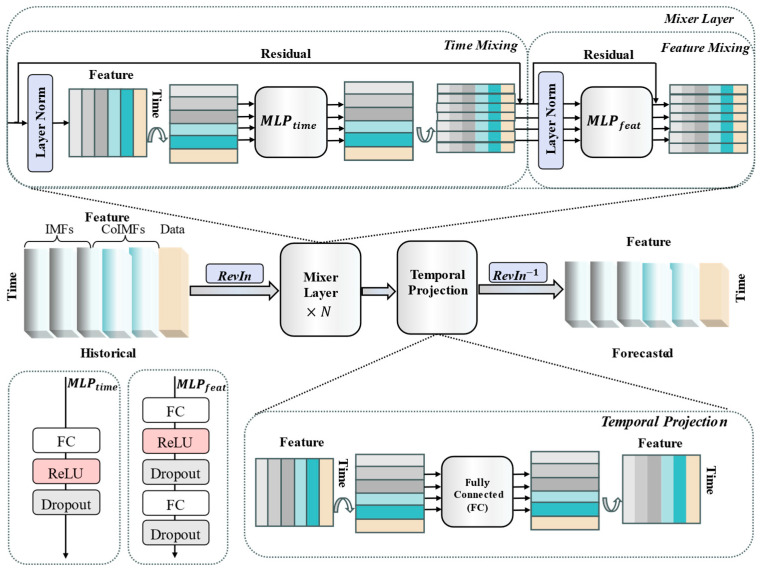
TSMixer model structure applied to gas prediction time-series data.

This paper adopts the reversible instance normalization (RevIN) method to improve the TSMixer model, mitigating the non-stationarity issue in methane concentration data and making it more suitable for methane concentration time-series prediction. The entire TSMixer framework is demonstrated, as shown in Figure 2. where after data input, reversible instance normalization is first performed, eliminating data distribution shifts and enhancing the model’s generalization ability; then, the RevIN output is directly used as input for the mixing layer, and the mixing layer calculations are performed continuously; subsequently, in the time projection layer, the input time length is extended to the prediction length through a fully connected layer linear mapping, followed by two transposition operations to maintain feature dimension consistency; and finally, the prediction values are restored to their original units through inverse normalization, facilitating result interpretation.

### 2.4. Anomaly Detection in Lonely Forest

In the context of monitoring coal and gas outburst anomalies, where data collection is limited or sample labeling is insufficient, semi-supervised or supervised monitoring methods are prone to poor model generalization due to insufficient training data. Isolation Forest (iForest) [27] is an unsupervised-learning anomaly detection method based on ensemble learning. This method constructs multiple binary isolation trees to quickly identify abnormal points in the feature space, achieving high detection accuracy and low time complexity, and can complete anomaly detection without relying on prior label information. iForest is based on the following assumptions: the number of abnormal data samples is small, and they can be isolated with a small number of steps. Through the selection of one of the extreme values of the selected features to partition the data points, the data space is recursively partitioned until all observations are isolated. The anomaly score formula of iForest is the core metric for measuring the degree of abnormality of data points, and its mathematical expression is

(22) $$s\left(x,n\right)=2^{-\frac{E\left(h\left(x\right)\right)}{c\left(n\right)}}$$

In Equation (22), $E\left(h\left(x\right)\right)$ is the average path length for sample *x* in all isolated trees; $c\left(n\right)$ is the normalization factor used to adjust the baseline value of the path length; and $s\left(x,n\right)$ is the anomaly score, with a value range of [0, 1]. When the score approaches 1, it is determined to be an anomaly.

### 2.5. Optimal Deployment of Gas Monitoring Sensors in Roadway Headings

The accurate monitoring of the methane volume fraction in the face can effectively prevent the occurrence of gas disasters, and the accuracy of methane volume fraction monitoring is largely affected by the arrangement of sensors [28]. Therefore, in the selected coal-mine face, the methane sensor arrangement is specially designed. The S1 sensor is suspended at the back of the face 5 meters away and moves forward with the advancement of the face. The S2 sensor is suspended 25 meters behind the face and monitors the volume fraction of gas in the return airflow, where the airflow is more stable. At the same time, the S3 sensor installed at the entrance of the roadway monitors the wind speed and methane concentration in the entire roadway.

**Figure 3:**
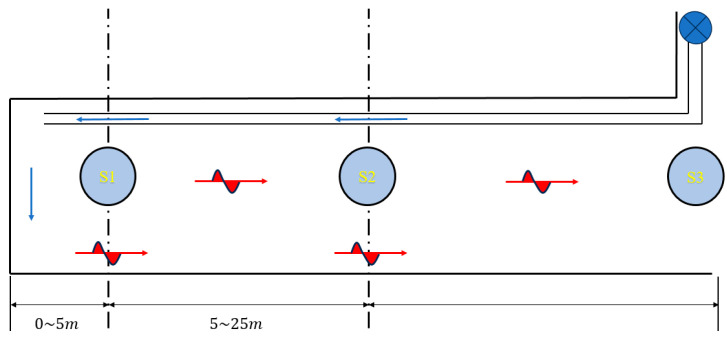
Schematic layout of drive surface sensors.

Figure 3 shows the deployment of the methane sensors in the roadway at the face of the excavation. The top right of the figure shows the local fan, which is installed in the fresh airflow tunnel near the entrance of the tunnel to provide power to the fresh airflow. The blue arrow represents the direction of the fresh airflow, and the red arrow represents the direction of the contaminated airflow. In the figure, the S1 sensor is located 5 m from the working face, the S2 sensor is located 25 m from the working face, and the S3 sensor is located at the entrance of the excavation roadway, which enables the comprehensive monitoring of the key parameters of the working face.

## 3. Results and Discussion

### 3.1. Data Collection and Preprocessing

This paper uses real coal-mine monitoring system data for experiments. The dataset synthesizes methane volume fraction data collected by gas sensors deployed at the face of a coal mine in Shaanxi Province, China, with a total of 2460 sampling points.

**Figure 4:**
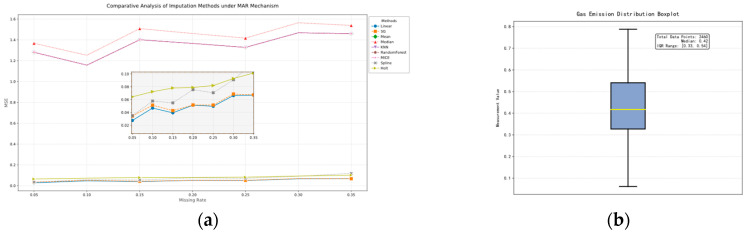
Comparison of different interpolation methods and box plots. (**a**) Interpolation method performance comparison under simulated random missing conditions with mean squared error metric. (**b**) Gas emission distribution analysis via box plot.

This paper selects the optimal data filling method by screening, using complete outflow monitoring data as the baseline dataset, and conducts interpolation method comparison experiments under simulated random missing conditions [29]. Nine typical methods, including Holt exponential smoothing, cubic spline interpolation, linear interpolation, Savitzky–Golay filtering interpolation, random forest interpolation, and k-nearest neighbor interpolation, were applied to artificially constructed missing samples. The accuracy of different methods was quantitatively evaluated using the mean squared error (MSE) metric. As shown in Figure 4a, linear interpolation exhibited the lowest mean squared error among the nine methods, demonstrating the best missing value recovery capability. Analysis indicated that this method effectively captured the temporal variation characteristics of the data, and its local linear approximation strategy based on adjacent observations better aligned with the variation patterns of this dataset. Based on this, linear interpolation was ultimately selected as the method for handling missing values.

When cleaning raw methane outburst data, the integrity of the data affects the identification of outliers. Therefore, linear interpolation is first applied to handle missing values. Subsequently, the global IQR (interquartile range) method is used to traverse the data after interpolating the missing values, and the identified outliers are also handled according to the missing value strategy, as shown in Figure 4b.

**Figure 5:**
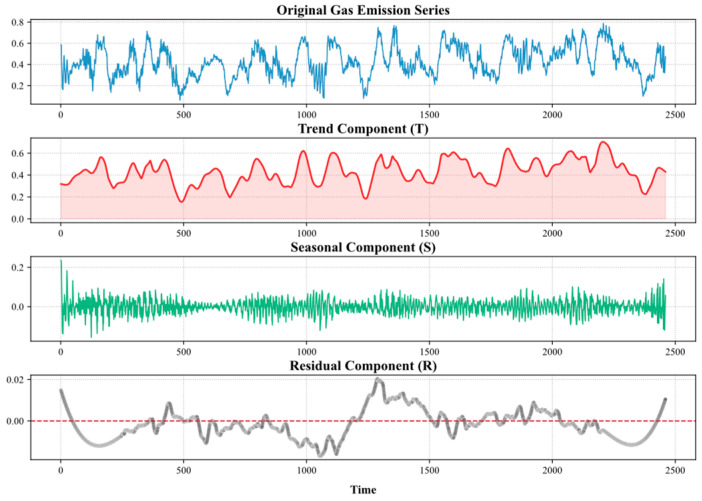
Methane outburst volume at coal-mining face after STL decomposition.

Based on Seasonal–Trend Decomposition using LOESS (STL), hierarchical decomposition is performed on the interpolated complete methane data, resulting in trend components, seasonal components, and residual components, as shown in Figure 5.

Trend terms are extracted using local weighted regression (LOESS) to identify the long-term evolution patterns of gas outburst rates. For example, as the tunneling face advances, gas release rates exhibit a nonlinear increasing trend over time. Seasonal terms utilize polynomial regression within a sliding window to capture fixed-period fluctuations, with parameters adjusted through iterative weighted least squares to suppress the influence of outliers on the decomposition results. Through the combination of trend terms and seasonal terms, most of the effective features can be extracted. It can be observed that methane outburst rates fluctuate between 0.2% and 0.6% during this time period, with significant periodic characteristics. Residual terms are smoothed using adaptive SG filtering, followed by sequence reconstruction, and the resulting smooth signal is used as input for the deep learning model.

### 3.2. Time Series Decomposition of Gas Emission Data

**Figure 6:**
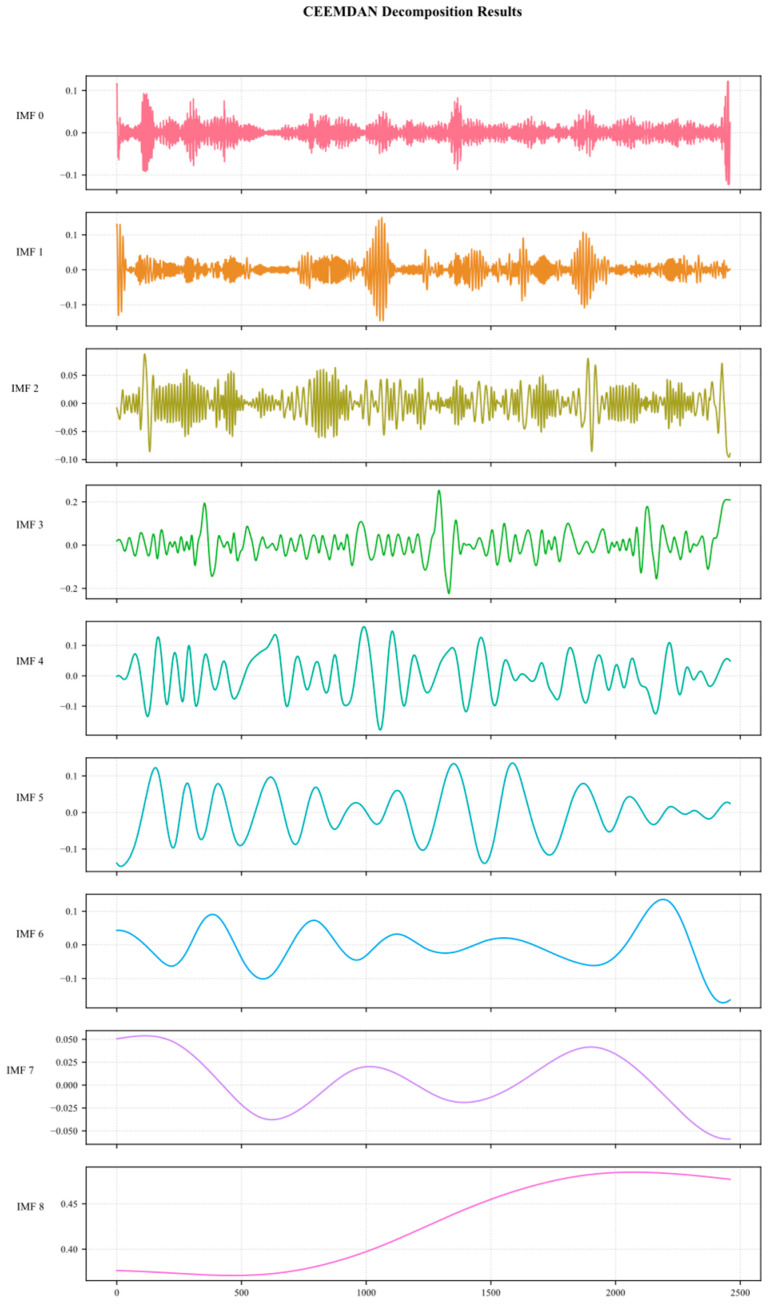
CEEMDAN decomposition plot.

After cleaning the raw methane outburst data, a smoothing method using STL decomposition and reconstruction was applied. Finally, the data were divided into multiple intrinsic mode function (IMF) components using CEEMDAN, representing the characteristics of the raw methane data at different time scales. The high-frequency components (IMF0-IMF2) typically characterize short-term fluctuations and noise, while the medium-frequency components (IMF3-IMF5) correspond to periodic patterns, and the low-frequency components (IMF6–IMF8) reflect trend-related characteristics. Figure 6 shows the decomposition results obtained using the CEEMDAN method.

**Figure 7:**
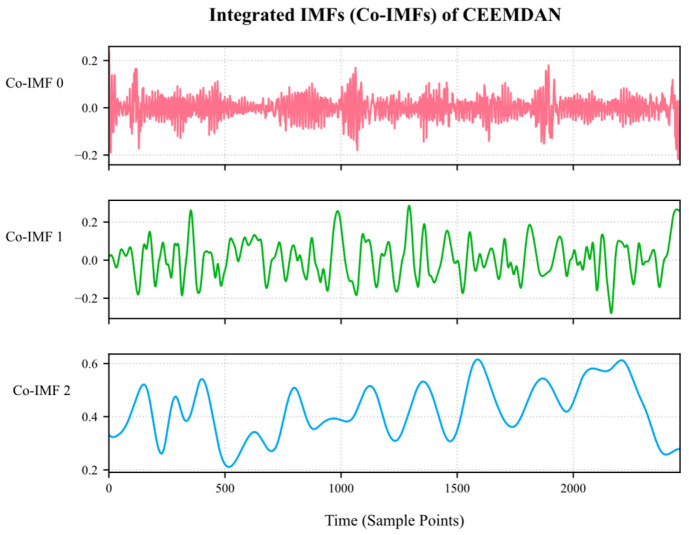
Plot of K-means clustering integration of IMF components.

The sample entropy values are calculated for each IMF component obtained from the decomposition of CEEMDAN, their complexity is quantified, and then K-means clustering is used to effectively integrate the complexity features of the IMF components, classifying the IMF into three categories: high, medium, and low frequency, as shown in Figure 7. Among these, the Co-IMF0 component consists of IMF0, IMF1, and IMF2; the Co-IMF1 component consists of IMF3 and IMF4; and the Co-IMF2 component consists of IMF5, IMF6, IMF7, and IMF8. The Co-IMF0, Co-IMF1, and Co-IMF2 components represent the high-, medium-, and low-frequency feature layers after K-means clustering.

**Figure 8:**
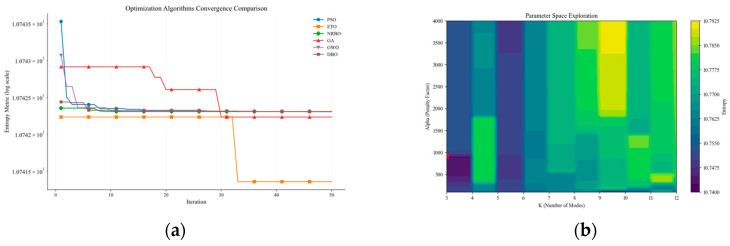
Plot of comparison of different algorithms.

For the high-frequency component Co-IMF0 obtained after k-means clustering, VMD is employed for secondary decomposition. To improve the decomposition performance of the VMD method, this paper employs the ETO algorithm, particle swarm optimization (PSO), and the Newton–Raphson-based optimizer (NRBO) to intelligently optimize the number of modal layers and penalty factors of VMD, and conducts comparative verification, as shown in Figure 8. The experiments were conducted under a unified benchmark setting: population size of 30, iteration limit of 50, modal number search range of [3, 12], penalty factor interval of [100, 4000], and sample entropy as the objective evaluation function. Figure 8a shows the fitness iteration curves of the ETO algorithm, NRBO algorithm, and PSO algorithm, and Figure 8b shows the parameter-space heat map. After the 33rd iteration, the ETO algorithm achieved a fitness value of 10.7415, making it the algorithm with the smallest fitness value. It could locate the global optimum with fewer iterations, demonstrating superior convergence speed and accuracy compared to the NRBO algorithm and PSO algorithm. It can be concluded that the ETO algorithm exhibits superior optimization performance.

**Figure 9:**
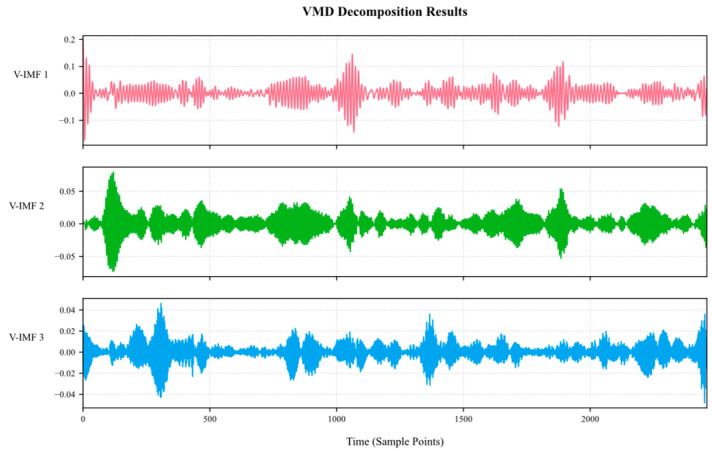
VMD decomposition plot.

Therefore, the ETO algorithm was used to perform a global search for the optimal solution in the parameter space to determine the optimal decomposition order that best fits the methane signal characteristics of the mine. Subsequently, VMD was used to decompose Co-IMF0 again, and the decomposition results are shown in Figure 9.

### 3.3. ETO of TSMixer Model

**Figure 10:**
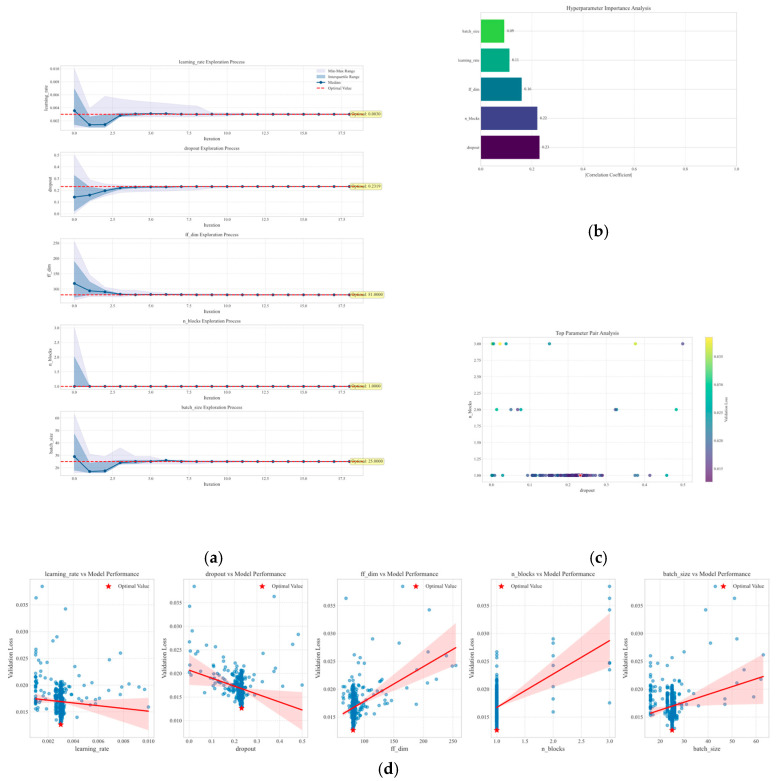
ETO algorithm for hyperparameter optimization of TSMixer model. (**a**) The exploration of different hyperparameters during the iteration process. (**b**) Hyperparameter importance ranking. (**c**) Impact of top hyperparameters on validation loss. (**d**) Hyperparameter optimization trajectory. (The red shaded area represents the region with the best model performance within different hyperparameter values.)

To avoid manual parameter tuning and further improve prediction accuracy, the first 60% of the original data were used for model optimization. The ETO algorithm was applied to optimize the hyperparameters of the TSMixer model, as shown in Figure 10. The optimization targets included the learning rate (0.001–0.01), dropout rate (0.0–0.5), feedforward network dimension (64–256), residual block number (1–4), and batch size (16–128). The mean squared error (MSE) parameter on the validation set was selected as the fitness metric, with 30 individuals selected and a maximum iteration count of 100. Figure 10a shows the exploration of different hyperparameters during the iteration process. The final optimal parameters are [‘learning_rate’:0.003003, ‘dropout’: 0.231887, ‘ff_dim’: 81, ‘n_blocks’: 1, ‘batch_size’: 25]. Figure 10b is a bar chart that ranks hyperparameters according to their correlation with model performance. Figure 10c visualizes the relationship between the two most important hyperparameters and the model validation loss using a scatter plot, and marks the optimal parameter combination. Figure 10d visualizes the exploration trajectory of each parameter during the hyperparameter optimization process, combining the distribution characteristics of the box plot and the dynamic changes in the trend line. The median line gradually approaches the optimal value line, and the distribution range continuously narrows, indicating that the parameter search is approaching stability.

### 3.4. Time-Series Prediction Model for Gas Emission Forecasting

This paper contains a total of 2460 sets of gas outburst data, with the training set, validation set, and test set accounting for 55%, 5%, and 40%, respectively. Due to the limited amount of historical data, using the model for long-term prediction would result in cumulative errors. Therefore, the intelligent prevention platform is used to obtain new data in real time and input it into the model to accurately predict future monitoring data.

**Figure 11:**
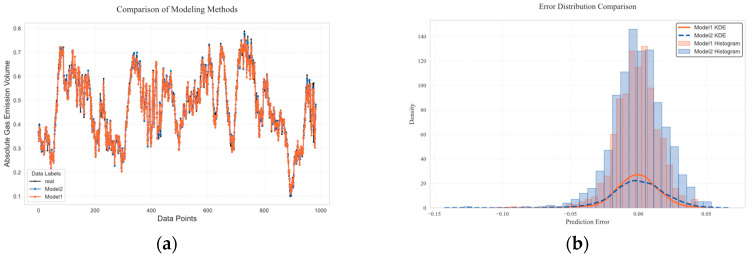
Comparison of prediction performance between different modeling methods. (**a**) Comparison of model predictions to actual data. (**b**) Kernel density estimation of prediction errors.

After time series decomposition, the TSMixer model is used to process multiple variables. All IMFs are jointly input, with each IMF treated as a feature channel. This avoids the problem of the separate modeling of each IMF, which fails to capture the potential correlations between different IMFs and leads to error accumulation. The model also fully learns the interaction information between components, effectively reducing modeling complexity and enhancing prediction accuracy. The subsequent experimental verification compares two modeling methods. To ensure fairness and consistency in the comparison experiments, the model hyperparameters were uniformly adjusted before testing. The figure shows the prediction curves for independent modeling and joint modeling methods, and the figure shows the prediction error distribution comparison. Model 1 represents the joint modeling prediction, and Model 2 represents the independent modeling prediction overlaid with the joint modeling prediction. In Figure 11a, it can be seen that Model 1 has the smallest prediction error and is closest to the actual data. The Kolmogorov–Smirnov test [30] shows that the distribution of prediction errors between the two models exhibits statistically significant differences (D = 0.0823, *p* = 0.0025). Since *p* < 0.05, we reject the null hypothesis (H_0_: the error distributions of the two models are identical) and conclude that the distribution differences are significant. Therefore, we visualize the results. In Figure 11b, the peak of the kernel density curve of Model 1 is located near the error value of 0.00, with a more concentrated peak density compared to Model 2, indicating that its prediction results are highly concentrated around the true value with minimal systematic bias. The peak of the kernel density curve of Model 2 is slightly shifted to the left toward the error value of −0.01, with the tail extending into a larger negative-error region, showing more pronounced data dispersion. Model 1 has a skewness close to 0, with error distribution exhibiting basic unimodal symmetry. In contrast, Model 2’s negative skewness indicates that errors are concentrated in the negative-value region, potentially indicating a systematic overestimation issue. Therefore, this study adopts the joint input of all IMF modeling methods.

**Figure 12:**
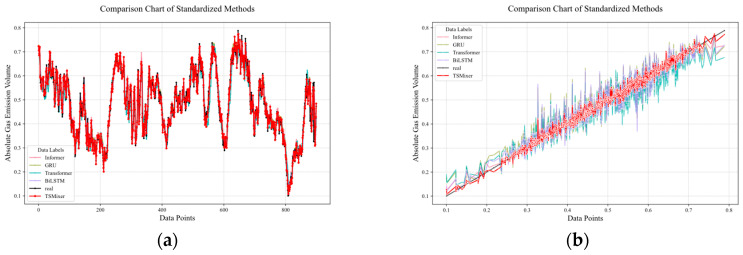
Comparison of prediction performance between different prediction models. (**a**) Comparative prediction accuracy of models in methane outburst forecasting. (**b**) diagonal error plot between different models.

GRU, Transformer, RNN, and BiLSTM were selected for training and compared with the TSMixer gas outburst prediction model. After training, the models were used to predict 900 sets of future data, and the results are shown in Figure 12. Figure 12a is a diagonal error plot, where the prediction results of each model are represented by points of different colors. The closer the line is to the black line, the smaller the prediction error. In the figure, it can be seen that the red line is closest to the actual data, indicating that the TSMixer model has the smallest prediction error. Figure 12b visually compares the prediction performance of each model at each time point, showing that the TSMixer model has a stronger ability to capture the sudden changes in methane outburst volume.

**Figure 13:**
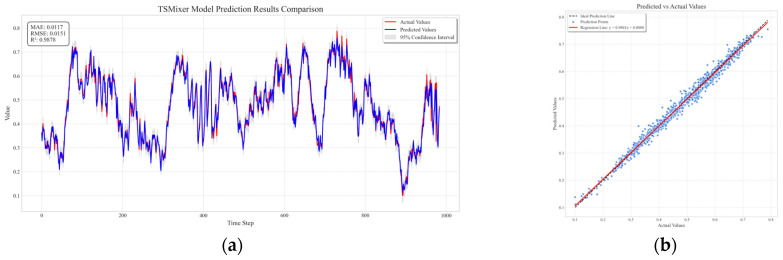
Comparison graph of predicted results and real data for the hybrid prediction model. (**a**) temporal evolution plot of the fusion model prediction results. (**b**) Scatter plot and regression line showing the fusion model’s prediction accuracy.

**Figure 14:**
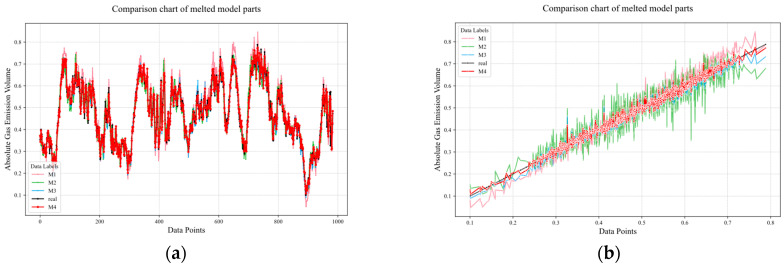
Comparison of ablation experiment prediction results and actual data. (**a**) temporal evolution plot of prediction results between different melted models. (**b**) diagonal error plot between different melted models.

Figure 13 shows the prediction performance of the fusion prediction model on a real dataset. This study is based on the TSMixer model framework and designs four sets of comparative experiments to explore the impact of different modules on model performance, as shown in Figure 14 and Table 1. The experimental settings are as follows: Experiment 1 (M1)—the TSMixer-RevIn architecture is used, and the CEEMDAN-VMD secondary decomposition module is removed to evaluate the contribution of this decomposition module to the overall performance. Experiment 2 (M2)—based on TSMixer-RevIn, the data smoothing processing module is removed to analyze the impact of data smoothing on model performance. Experiment 3 (M3)—the TSMixer base model is used without the reversible normalization improvement to investigate the extent to which reversible normalization enhances model performance. Experiment 4 (M4)—as a control group, the complete TS Mixer-RevIn model is used, retaining all modules, to provide a baseline performance comparison. Through ablation experiments, we systematically analyzed the specific impact of each module on the performance of the TSMixer model, providing a solid experimental basis for subsequent model optimization and improvement.

### 3.5. Unsupervised Early-Warning Model for Time Series

After the prediction is completed, the predicted future time data are used as input for STL decomposition. The residuals and original methane outburst data obtained after decomposition are standardized using Z-scores to eliminate unit differences. Separate Solitary Forest models are then constructed for them. A slightly higher contamination parameter is applied to the residual model to enhance sensitivity to residual anomalies. Any model labeled as anomalous is considered anomalous. Finally, the anomaly scores from the two models are weighted-averaged (original data weight: 0.6; residual weight: 0.4) to evaluate the severity of anomalies, resulting in the category of methane state at future time points. The isolated forest model based on original data is used to detect global anomalies, while the residual-based isolated forest model focuses on short-term fluctuation anomalies, effectively avoiding misjudgments caused by global trends or periodic fluctuations and capturing anomaly patterns from different perspectives to complement each other.

**Figure 15:**
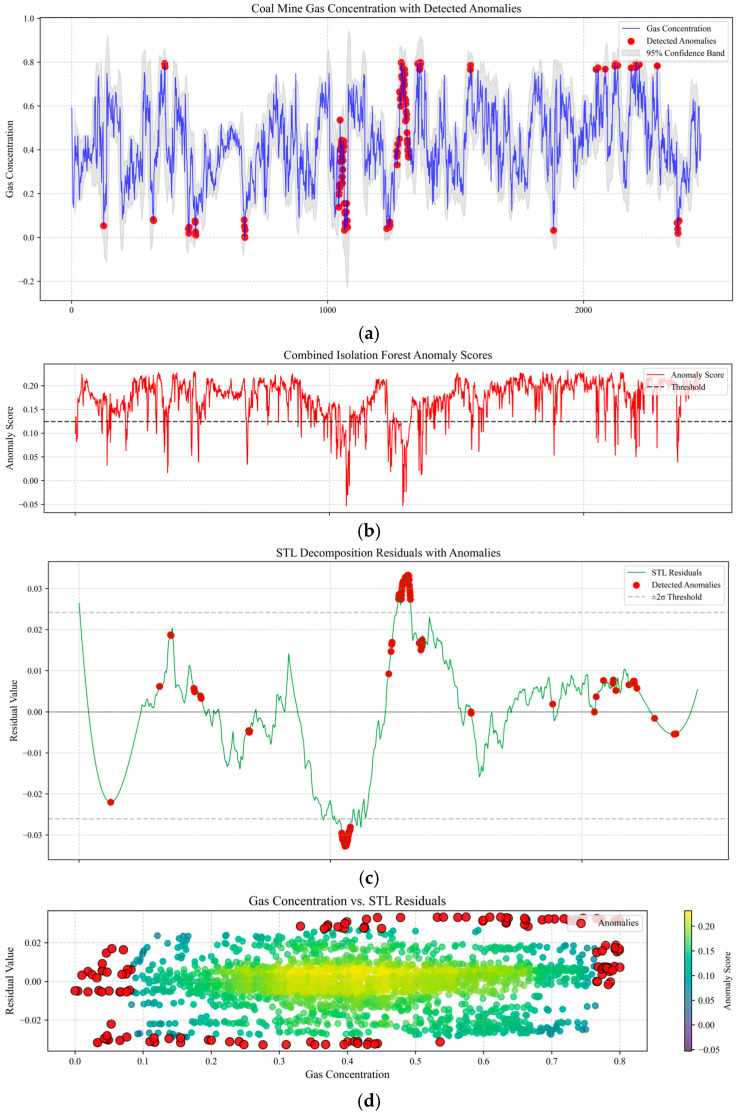
Real-time gas emission volume mutation point detection graph. (**a**) Original methane concentration time series with detected anomalies and confidence interval. (**b**) Composite anomaly score time series with dynamic threshold optimization. (**c**) STL decomposition residual analysis with ±2σ thresholds and transient disturbance detection. (**d**) Scatter plot of methane concentration vs. STL residuals with composite anomaly characterization.

Next, we validated the effectiveness of the methane concentration anomaly detection model through data visualization. Figure 15a shows the original methane concentration time series and marked anomaly points, where the blue time series curve reflects the dynamic fluctuations in methane concentration, the red scatter points mark the global anomalies detected by the isolated forest, and the gray shaded area represents the 95% confidence interval. Anomalies are concentrated in the high-value region where concentrations exceed 0.6 and are predominantly located outside the confidence interval, confirming that the global model is sensitive to extreme values deviating from the main distribution. Figure 15b shows the combined anomaly score time series and threshold lines. The red broken line represents the weighted fusion of the comprehensive anomaly scores, while the gray dashed line denotes the dynamically optimized threshold line obtained through grid search. A sustained threshold violation occurs around t ≈ 1300, corresponding to the potential risk in Figure 15a where concentration suddenly increases but remains within the confidence interval, demonstrating the weighted strategy’s ability to detect hidden anomalies. Figure 15c shows the STL decomposition residual time series and marked anomaly points, accompanied by ±2σ threshold lines. In a comparison with Figure 15a, it is found that the residual anomaly points (marked in red) mostly correspond to transient disturbances during equipment operation (such as sensor noise or sudden changes in local airflow) rather than systemic failures. For example, at t ≈ 1100, the residuals exhibit significant oscillations (amplitude > 0.02) while the original concentration remains close to the trend line, indicating that the residual model is more sensitive to small-scale local disturbances than the global model. Figure 15d shows a scatter plot of methane concentration versus STL residuals, with colors indicating anomaly scores. The color mapping demonstrates that anomaly scores increase gradually from the center toward the edges, validating the weighted fusion strategy’s ability to characterize composite anomalies.

## 4. Conclusions

This paper proposes a hybrid analysis method that integrates temporal prediction and unsupervised anomaly detection, focusing on solving the problem of real-time methane monitoring in coal-mine tunneling workfaces. In the field of temporal prediction, an innovative composite architecture is constructed by combining a data smoothing method based on STL decomposition, adaptive SG filtering, and reconstruction with a TSMIxer improved by reversible instance normalization. Through the joint modeling of the IMFs generated by secondary decomposition, model performance is significantly improved. For anomaly detection tasks under conditions where anomaly samples are scarce, this paper constructs a novel unsupervised anomaly identification framework based on STL decomposition and a multi-scale lonely forest, providing a new monitoring method for coal-mine tunneling workface engineering.

This study combines computer experiments and theoretical analysis to conduct an in-depth investigation of methane dynamic prediction and anomaly detection technologies. The main research results are reflected in the following three aspects:

(1) A hybrid prediction model based on feature decomposition was constructed, and the innovatively introduced STL decomposition–adaptive SG filtering–reconstruction smoothing method is more suitable for methane concentration nonlinear fluctuation scenarios. A CEEMDAN-Kmeans-VMD secondary decomposition strategy was proposed, utilizing the K-means algorithm to cluster and integrate IMFs through sample entropy, thereby enhancing the model’s feature extraction capability. The ETO algorithm was employed to optimize VMD, effectively addressing the challenge of determining the number of modes in VMD.

(2) An improved TSMixer model based on IMF joint modeling was innovatively proposed. Reversible instance normalization was used to eliminate data distribution bias and enhance the model’s generalization ability, and the ETO algorithm was employed to achieve the intelligent optimization of model hyperparameters. In leveraging the multi-input processing capability of the TSMixer model, all IMFs are jointly processed without losing the interaction information of each component, thereby avoiding the accumulation of errors from separate IMF modeling. This effectively reduces modeling complexity and enhances prediction accuracy.

(3) An abnormal methane outburst detection method based on STL decomposition and dual isolated forest collaboration has been established. Through a complementary mechanism between global trends and short-term fluctuations, the accuracy and robustness of anomaly detection have been effectively improved. Under conditions of insufficient abnormal samples, the reliable detection of sudden increase features has been achieved, providing a new technical approach for practical engineering applications.

This paper provides an innovative monitoring solution for coal-mine gas disaster prevention and control, which helps improve the timeliness of risk warnings and reduce the incidence of production safety accidents. Follow-up research will integrate acoustic emission monitoring data with theoretical modeling methods to establish a multimodal real-time monitoring indicator system and continuously optimize intelligent monitoring methods to promote the intelligent transformation and upgrading of the coal industry.

## Figures and Tables

**Table 1:** Error between predicted results and real values for ablation experiments.

Model	RMSE	MAE	MSE	$R^{2}$
M1	0.024490	0.018685	0.000599	0.977127
M2	0.029455	0.022865	0.000867	0.953183
M3	0.018559	0.014061	0.000344	0.981404
**M4**	**0.015060**	**0.011677**	**0.000226**	**0.987754**

## Data Availability

The data are not publicly available due to commercial confidentiality, as they contain information that could compromise the privacy of research participants.
